# Orai1 inhibitor STIM2β regulates myogenesis by controlling SOCE dependent transcriptional factors

**DOI:** 10.1038/s41598-019-47259-5

**Published:** 2019-07-25

**Authors:** Kyu Min Kim, Anshul Rana, Chan Young Park

**Affiliations:** 1 Department of Biological Sciences, School of Life Sciences, UNIST, Ulsan, 44919 Republic of Korea; 2 Department of Molecular and Cellular Physiology, Stanford University School of Medicine, Stanford, CA 94305 USA

**Keywords:** Calcium signalling, Differentiation

## Abstract

Store-operated Ca^2+^ entry (SOCE), the fundamental Ca^2+^ signaling mechanism in myogenesis, is mediated by stromal interaction molecule (STIM), which senses the depletion of endoplasmic reticulum Ca^2+^ stores and induces Ca^2+^ influx by activating Orai channels in the plasma membrane. Recently, STIM2β, an eight-residue-inserted splice variant of STIM2, was found to act as an inhibitor of SOCE. Although a previous study demonstrated an increase in STIM2β splicing during *in vitro* differentiation of skeletal muscle, the underlying mechanism and detailed function of STIM2β in myogenesis remain unclear. In this study, we investigated the function of STIM2β in myogenesis using the C2C12 cell line with RNA interference-mediated knockdown and CRISPR-Cas-mediated knockout approaches. Deletion of STIM2β delayed myogenic differentiation through the MEF2C and NFAT4 pathway in C2C12 cells. Further, loss of STIM2β increased cell proliferation by altering Ca^2+^ homeostasis and inhibited cell cycle arrest mediated by the cyclin D1-CDK4 degradation pathway. Thus, this study identified a previously unknown function of STIM2β in myogenesis and improves the understanding of how cells effectively regulate the development process via alternative splicing.

## Introduction

Myogenesis is the process by which somatic cells form mature muscle tissue through a series of complex regulated processes of proliferation, cell cycle arrest, and differentiation. Numerous signaling pathways help to precisely control these stages^1,2^. The mitogen-responsive signal then degrades cyclin D1 and its catalytic partner CDK4 to induce exit of the cell from the G1 phase to enter the G0 phase, a quiescent post-mitotic state^3,4^. Further, myogenin acts with the myocyte enhancer factor 2 (MEF2) transcription factor to regulate expression of the late myogenesis marker genes such as myosin heavy chain (*MHC*), which is usually expressed after the fusion of myoblasts into myotubes. In particular, MEF2C protein, which was first identified in the nuclei of myotube cells^5^, is a main regulator of muscle development^6–8^.

Store-operated Ca^2+^ entry (SOCE), orchestrated by the two key mediators stromal interaction molecule (STIM) and Orai, is involved in numerous cellular processes, including secretion, muscle contraction, cell growth, and muscle development^9,10^. STIM is a single-transmembrane Ca^2+^ sensor located in the endoplasmic reticulum (ER)^11–13^, whereas Orai is a plasma membrane-located Ca^2+^ channel with four transmembrane domains that harbor the pore subunit of SOCE^14–16^. Many studies have demonstrated that STIM1-dependent Ca^2+^ signaling is crucial for the onset of skeletal muscle development^17,18^. In addition, STIM2 regulates the intracellular Ca^2+^ distribution and myogenesis^19,20^.

SOCE modulates the activity of various Ca^2+^-dependent enzymes that regulate the myogenesis-related transcription factors, including nuclear factor of activated T cells (NFAT) that plays a regulatory role in skeletal muscle development^21^. *In vivo*, mice lacking NFAT4 showed impaired muscle development during embryogenesis^22^. *In vitro*, a calcineurin/NFAT4 pathway was found to regulate myogenin induction, and efficient induction and progression of myogenesis required the positive feedback between STIM1 and NFAT4^23,24^.

There are many potential mechanisms by which SOCE is regulated, such as internalization and phosphorylation, and alternative splicing. The recently identified spliced isoform of STIM2, STIM2β (also known as STIM2.1), is highly conserved and acts as a potent inhibitor of SOCE^25,26^. STIM2β contains an additional eight amino acids in CRAC activation domains (CADs; also known as the STIM1 Orai1 activation region [SOAR] or CCb9)^27–30^ and forms a heterodimer with other STIM isoforms to inhibit Ca^2+^ release-activated Ca^2+^ (CRAC) channels through an allosteric mechanism^31^. Through this alternative splicing of STIM2, the cells can effectively control the Ca^2+^ homeostasis by preventing Orai1 channel cross-linking^32^ and regulate the balance between SOCE activators and inhibitors. However, the physiological role and detailed underlying mechanism of STIM2β remain unclear.

Accordingly, in this study we explored the detailed function of STIM2β in myogenesis using C2C12 cells with STIM2β knockdown or knockout approaches. We explored the effects of STIM2β loss or deficiency on the key regulators of myogenesis described above, including the NFAT4 and MEF2C signaling pathway, along with its effects on Ca^2+^ homeostasis and cell cycle arrest via analysis of changes in the expression of cyclin D1 and CDK4. Our subsequent molecular mechanistic study suggests that the STIM2β signaling pathway serves as a switch cell fate between proliferation and skeletal muscle differentiation. These results can provide new insight into the molecular interactions and roles of splicing events in the regulation of skeletal development with potential implications for regenerative medicine.

## Results

### Splicing form of STIM2β increased during myogenesis

**Figure 1:**
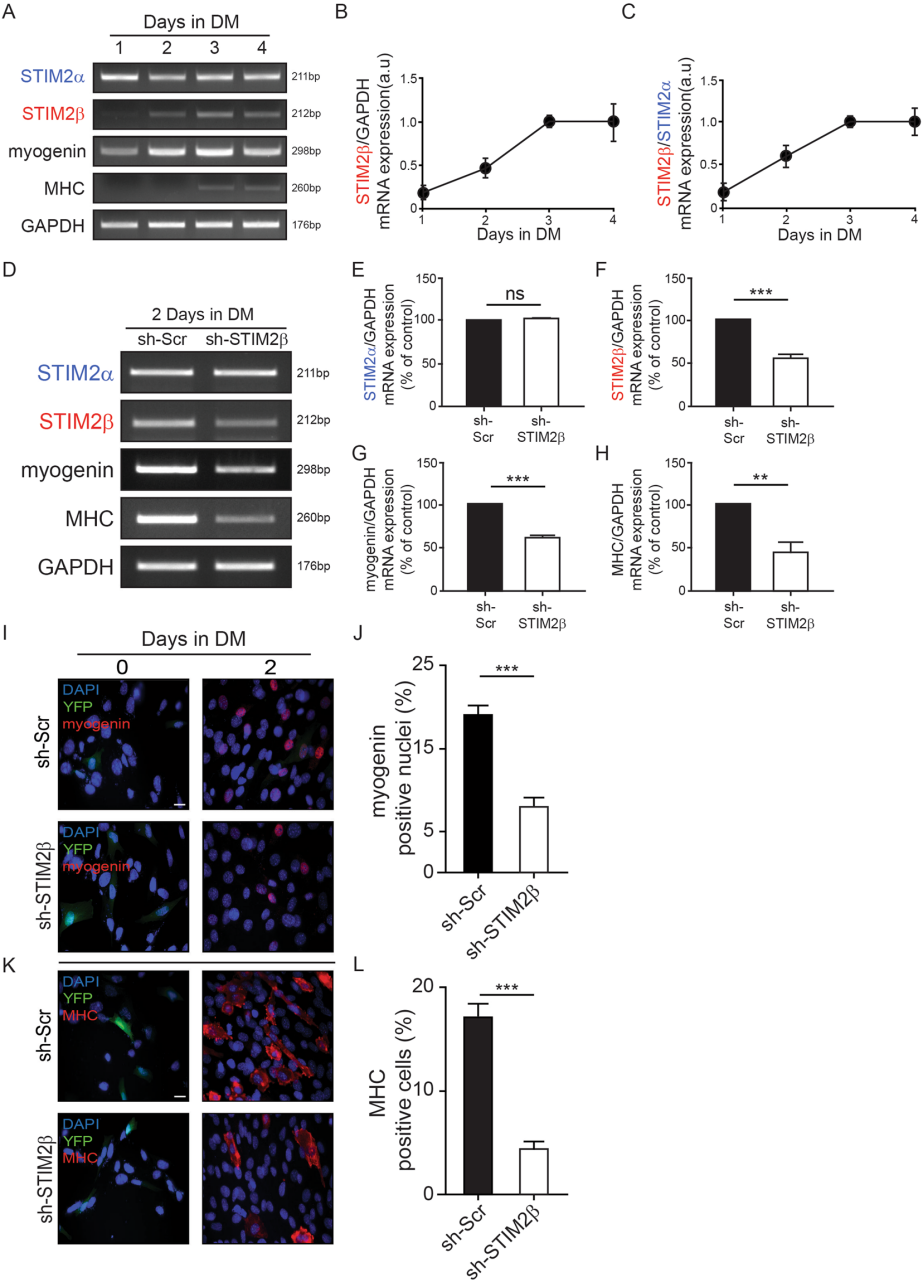
STIM2 splicing affects myogenesis. (**A**) Expression of STIM2 splicing variants during the differentiation of C2C12 myoblast cells (**B**) GAPDH-normalized expression levels of STIM2β during C2C12 myoblasts differentiation. (**C**) Histogram of the STIM2α/STIM2β ratio during C2C12 differentiation. (**D**) Polymerase chain reaction analysis of cDNA from Control (sh-Scr) and STIM2β knock-down (sh-STIM2β) cells 2 day after inducing differentiation. (**E**–**H**) GAPDH-normalized expression levels of STIM2α (**E**), STIM2β (**F**), myogenin (**G**) and MHC (**H**). (**I**,**J**) The fraction of myogenin positive cells from control cells (**J**, *black bar)* and STIM2β knock-down cells (**J**, *white bar*). (n > 80 cells for each group). (**K**,**L**) The fraction of MHC positive cells from control cells (**L**, *black bar)* and STIM2β knock-down cells (**L**, *white bar*). (n > 80 cells for each group). Error bars show means ± SEM. (All scale bars, 20 μm.) The results are representative of at least three independent experiments.

We first investigated the mRNA expression levels of STIM2α and STIM2β during C2C12 myoblast cells differentiation. C2C12 myoblasts were cultured in a differentiation medium and the mRNA expression level was assessed daily using quantitative PCR analysis. The STIM2β expression level showed an approximately 5-fold increase after induction of differentiation (Fig. 1A–C)^26^, whereas the mRNA levels of STIM2α and other SOCE components showed no dramatic change during myogenesis (Figs 1A and S1A). Therefore, we further explored the function of STIM2β during myogenesis.

### Knockdown of STIM2β delayed myogenesis

To identify the function of STIM2β in C2C12 myogenesis, we knocked down STIM2β using specific shRNA in C2C12 myoblasts. The specificity of the sh-STIM2β expression construct was confirmed, which effectively knocked down only STIM2β expression (>40%) but not STIM2α and other SOCE components expression in cells during differentiation (Figs 1D–F and S1A,B). Interestingly, STIM2β knockdown also caused significant suppression of the mRNA expression levels of the myogenesis marker genes myogenin and MHC by 40% and 50%, respectively, compared with control cells expressing sh-scramble after inducing differentiation for two days (Fig. 1D–H).

To further validate the function of STIM2β in myogenesis, we investigated the population of myogenin- and MHC-positive cells at day 2 after inducing differentiation following transfection of sh-Scr or sh-STIM2β. The C2C12 cells transiently expressing sh-STIM2β showed about 50% of reduced myogenesis compared to sh-Scr treated cells at differentiation day 2 (Fig. 1I–L). These results indicated that transient knockdown of STIM2β suppressed the expression of myogenic genes during C2C12 myogenesis.

Collectively, these results demonstrated that the splicing of STIM2 dramatically changed during myogenesis and that STIM2β regulates the expression of myogenic genes. However, the sh-RNA knockdown approach had a short lifespan to checked longer time period (Fig. S1C). Given the limitation of this approach for further exploration of C2C12 myogenesis, we further established STIM2β knockout cells using CRISPR-Cas9 technology.

### STIM2β knockout cell line generated by CRISPR-Cas9

To generate the STIM2β knockout C2C12 myoblast cell line using the CRISPR-Cas9 gene editing system, we first designed sgRNA targeting the exon 9 region, which only includes STIM2β. After 4-day treatment of hygromycin for selecting the genome-edited cells, we observed an evident decrease in the length of the PCR product, and confirmed the 226-nucleotide deletion around the exon 9 region by sequencing (Fig. S1A). To test whether this CRISPR/Cas9-mediated deletion successfully knocked out the exon9 region which included only in STIM2β, we evaluated the expression level of the STIM2 splicing variants STIM2α, STIM2β and other SOCE components after inducing myogenesis. The C2C12 myoblasts showed up-regulation of STIM2β expression during myogenesis, whereas the expression of STIM2β was completely abolished in the CRISPR-Cas9-mediated exon 9-deleted cells. In contrast, the expression level of STIM2α and other SOCE components did not show a significant difference between wild-type and STIM2β-knockout cells (Fig. S1D–G), indicating that STIM2β-knockout cells were successfully generated by the CRISPR-Cas9 system targeting exon 9.

### STIM2β knockout cells showed delayed expression of myogenic genes

**Figure 2:**
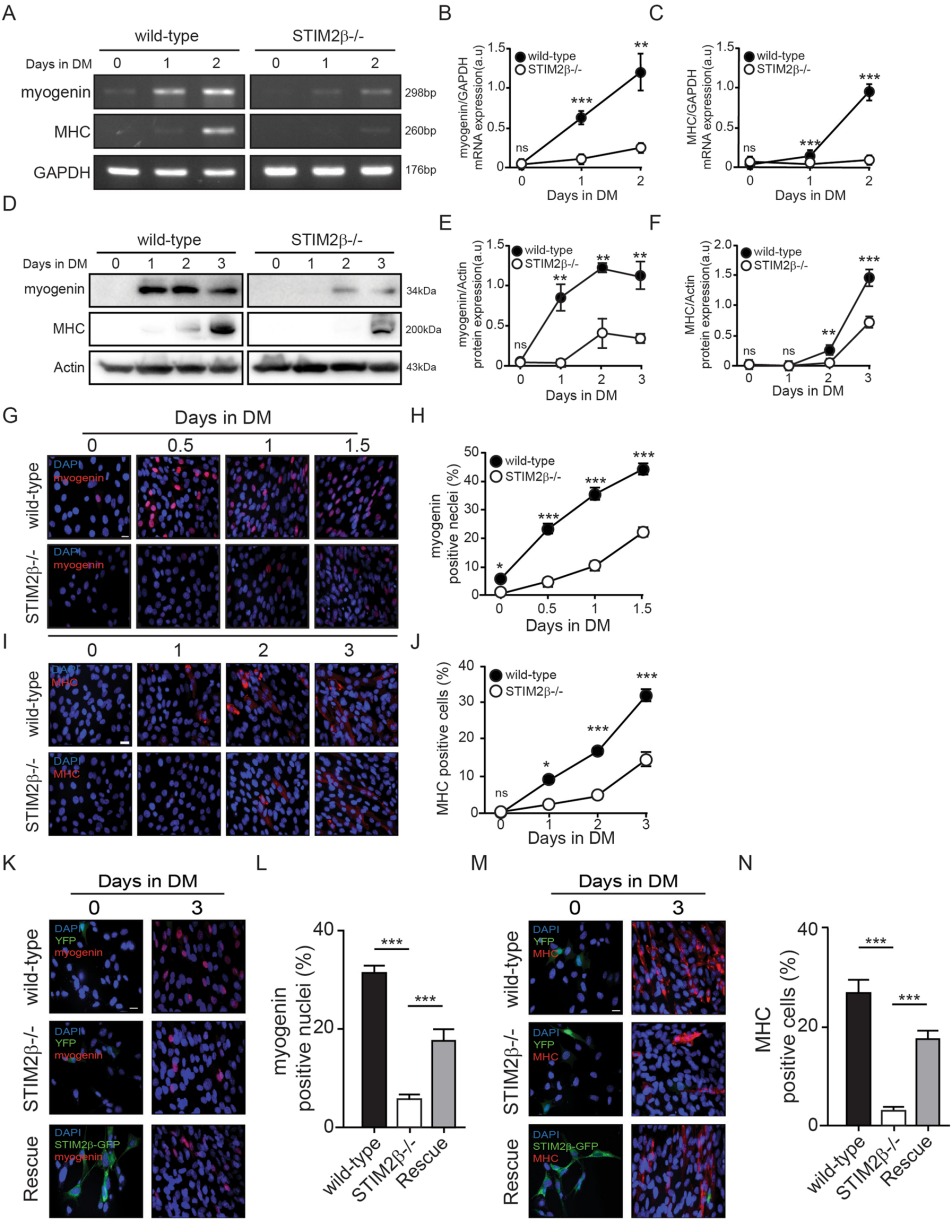
STIM2β knockout delayed C2C12 myoblast differentiation. (**A**) Comparative myogenin and MHC mRNA expression by RT-PCR between wild-type and STIM2β knockout cells. (**B**,**C**) GAPDH-normalized expression levels of myogenin (**B**) and MHC (**C**) of wild-type cells (Black circle) and STIM2β knockout cells (White circle) during myogenesis. (**D**) Immunoblot analysis of wild-type cells and STIM2β knockout cells to measure the protein expression of myogenin and MHC during myogenesis. (**E**,**F**) Actin-normalized expression levels of Myogenin (**E**) and MHC (**F**) of wild-type cells (Black circle) and STIM2β knockout cells (White circle). (**G**) Immunofluorescence staining of myogenin protein during the differentiation of wild-type cells (top) and STIM2β knockout cells (bottom). (**H**) Myogenin positive nuclei population of wild-type cells (Black circle) and STIM2β knockout cells (White circle). (n > 80 cells for each group) (**I**) Immunofluorescence staining of MHC protein during the differentiation of wild-type cells (Top) and STIM2β knockout cells (Bottom). (**J**) The fraction of MHC positive cells of wild-type cell (Black circle) and STIM2β knockout cells (White circle). (n > 80 cells for each group) (**K**–**N**) Immunofluorescence staining of myogenin (**K**,**L**) and MHC (**M**,**N**) protein during the differentiation of wild-type cells (Top, black bar), STIM2β knockout cells (Middle, White bar) and transiently STIM2β expressed STIM2β knockout cells (Bottom, gray bar). (n > 80 cells for each group). Error bars show means ± SEM. (All scale bars: 20 μm.) The results are representative of at least three independent experiments.

Given that the results described above strongly implicated a role of STIM2β in C2C12 myogenesis, we next measured the expression of myogenic marker genes in both wild-type and STIM2β knockout cells after inducing myogenesis. The GAPDH*-*normalized mRNA expression levels of both myogenin and MHC were significantly decreased in STIM2β knockout cells compared to those of wild-type cells (Fig. 2A–C). Although the relative expression level of myosin increased during the 2-day differentiation period, reaching a level approximately one-fifth that of wild-type C2C12 myoblast cells (Fig. 2A,B), there was no such increase in MHC mRNA expression levels after differentiation induction, while the expression level in wild-type cells was significantly increased during differentiation (Fig. 2A,C).

Western blot analysis confirmed these results at the protein level, in which STIM2β knockout cells showed significantly decreased protein expression levels of myogenesis marker genes. Myogenin was detected in the lysate of wild-type C2C12 myoblast cells as of 1 day after inducing myogenesis, but was only weakly detected in the lysate of STIM2β knockout cells with no tendency to increase during differentiation progression (Fig. 2D,E). Moreover, the MHC protein expression was strongly inhibited in STIM2β knockout cells, and was only weakly detected as of day 3 of differentiation, whereas MHC protein was clearly expressed as of 1 day after inducing myogenesis in wild-type cells and increased with the progress of differentiation (Fig. 2D,F). These results indicated that the expression of key myogenic genes was delayed in STIM2β knockout cells.

To further validate the delayed myogenesis of STIM2β knockout cells, we investigated the fraction of myogenic gene-positive nuclei among wild-type and STIM2β knockout cells. Immunofluorescence staining data showed that both myogenin (Fig. 2G,H) and MHC (Fig. 2I,J)-positive cells frequencies were decreased by about half in STIM2β knockout cells compared with those of the wild-type cells. These results further support the important role of STIM2β in regulating the expression of myogenic genes.

Moreover, after transiently expressing STIM2β in STIM2β knockout cells, immunofluorescence staining at 3 days after inducing differentiation showed that the fractions of myogenin-positive (Fig. 2K,L) cells and MHC-positive cells increased markedly (Fig. 2L,M). This observation indicated that the delayed expression of myogenic genes of STIM2β knockout cells was derived from the interference of STIM2β signaling.

### STIM2β plays a role in NFAT4 and MEF2C-mediated myogenesis

**Figure 3:**
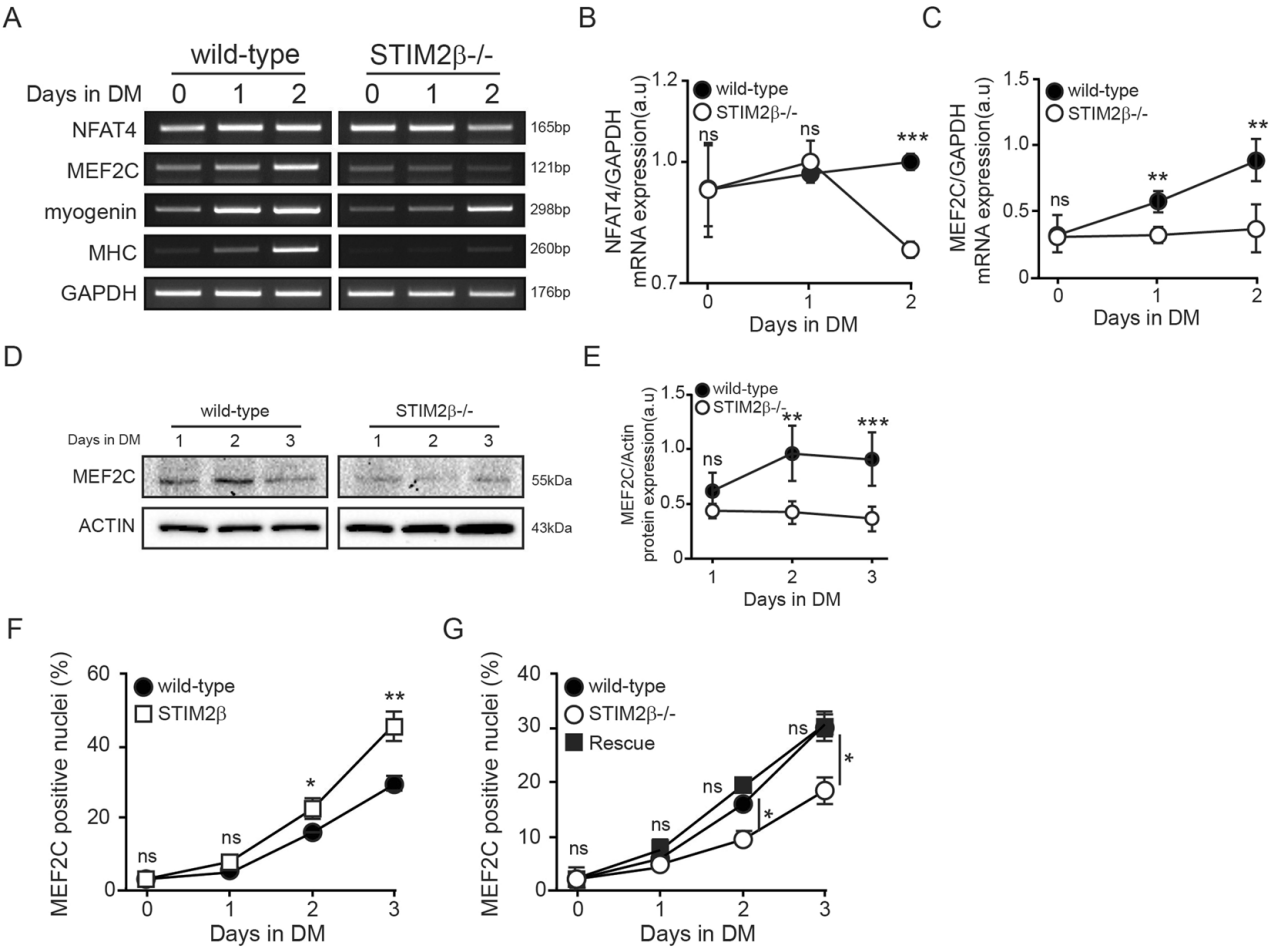
STIM2β knockout inhibited NFAT4 and MEF2c. (**A**) Comparative NFAT4 and MEF2c mRNA expression by RT-PCR between wild-type and STIM2β knockout cells. (**B**,**C**) GAPDH-normalized expression levels of NFAT4 (**B**) and MEF2c (**C**) of wild-type cells (Black circle) and STIM2β knockout cells (White circle) during myogenesis. (**D**) Immunoblot analysis of wild-type cells and STIM2β knockout cells to measure the protein expression of MEF2C during myogenesis. (**E**) Actin-normalized expression levels of MEF2C in wild-type cells (Black circle) and STIM2β knockout cells (White circle). (**F**) The fraction of MEF2c positive nuclei of wild-type cell (Black circle) and STIM2β over-expressed cells (White rectangular). (n > 60 cells for each group) (**G**) The fraction of MEF2c positive nuclei of wild-type cell (Black circle), STIM2β knockout cells (White circle) and transiently STIM2β expressed STIM2β knockout cells (Black rectangular). (n > 60 cells for each group). Error bars show means ± SEM. The results are representative of at least three independent experiments.

We next compared the well-known Ca^2+^-related myogenesis regulator genes, *NFAT4* and *MEF2C*, between wild-type and STIM2β knockout cells during myogenesis. First, to test whether STIM2β knockout could induce a downstream Ca2+ signaling, we introduced GFP-NFAT4 into wild-type and STIM2β knockout cells. Before inducing differentiation, wild-type and STIM2 knockout cells showed similar nuclear translocation of GFP-NFAT4 (Fig. S2). Unexpectedly, however, the mRNA expression level of *NFAT4* rapidly decreased after inducing differentiation in STIM2β knockout cells but gradually increased in wild-type cells (Fig. 3A,B).

Notably, the mRNA expression of MEF2C in STIM2β knockout cells failed to increase even after inducing differentiation (Fig. 3A,C). We performed Western blot experiment of MEF2C. As expected, the expression pattern of MEF2C protein was different in both cells after differentiation. The wild type cells showed an increased expression level of MEF2C. However, the protein expression level of MEF2C was not changed in the STIM2β knockout cells after differentiation. It is consistent with the result of no change in the mRNA expression level of MEF2C in Stim2β knockout cells (Fig. 3D,E). Furthermore, 3 days after inducing differentiation, MEF2C-positive nuclei increased from 30% to 45% when transiently overexpressed STIM2β (Fig. 3F), and the significant decrease in MEF2C-positive cells from STIM2β knockout was rescued by transient expression of STIM2β (Fig. 3G). Taken together, these lines of evidence collectively suggest that STIM2β positively regulates myogenesis by activating the MEF2C and NFAT4 signaling pathway.

### STIM2β regulates myoblast proliferation via Ca^2+^ homeostasis

**Figure 4:**
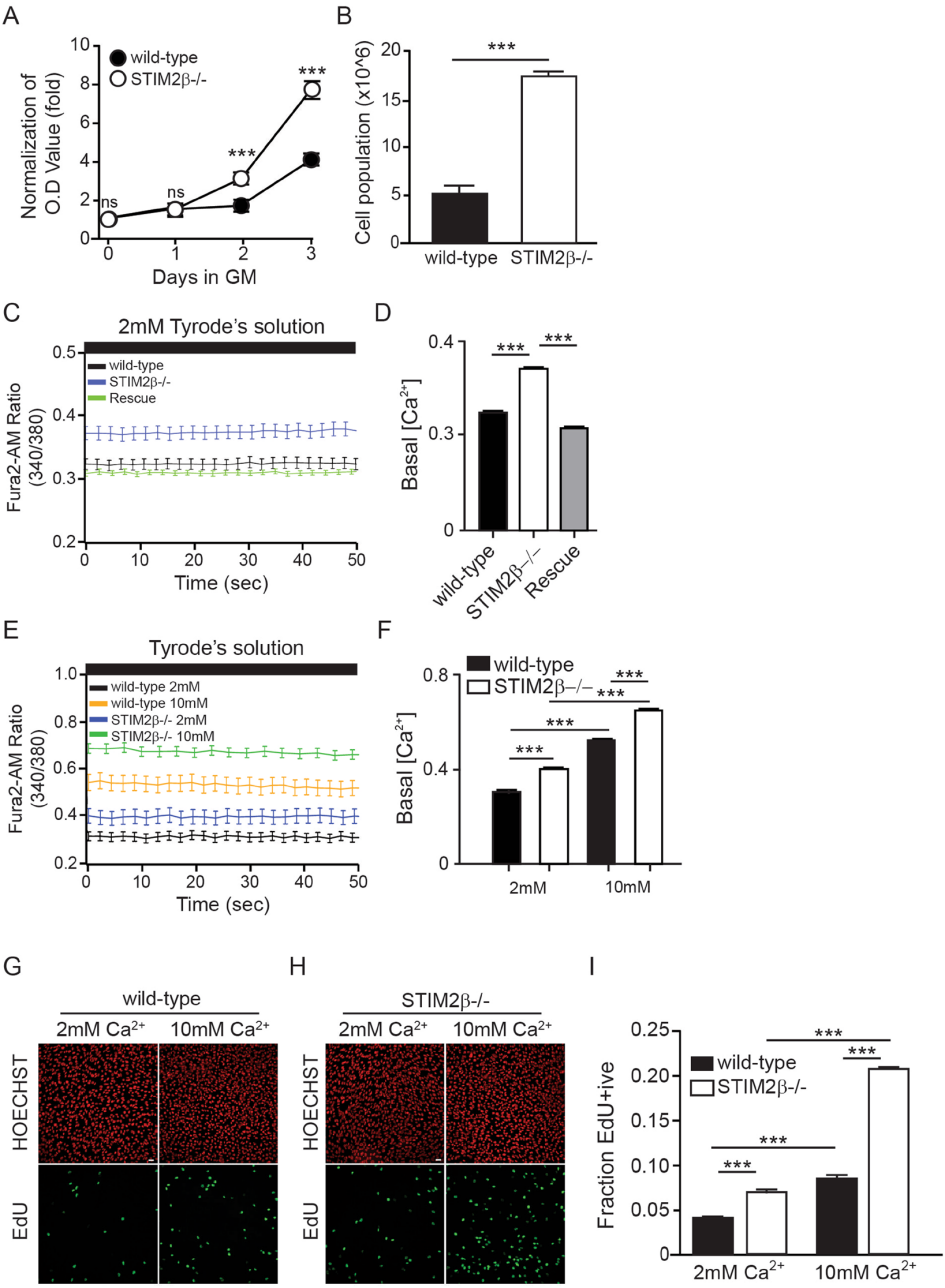
STIM2β knockout promoted proliferation via Ca^2+^ homeostasis in C2C12 myoblast cells. (**A**) MTT Proliferation assay of wild-type cells (Black circle) and STIM2β knockout cells (White circle). (n = 4 respective experiment for each group) (**B**) Histogram of cell population of wild-type cells (Black bar) and STIM2β knockout cells (White bar). (**C**) Fura-2 Ca^2+^ measurements in 2 mM Ca^2+^ Tyrode’s solution. wild-type (black), STIM2β knockout cells (blue) and transiently STIM2β expressed in STIM2β knockout cells (green). (**D**) Histogram of basal calcium level of wild-type (black), STIM2β knockout cells (white) and transiently STIM2β expressed in STIM2β knockout cells (gray). (n > 40 cells for each group) (**E**) Fura-2 Ca^2+^ measurements in Tyrode’s solution. wild-type (black), STIM2β knockout cells (blue) in 2 mM extracellular Ca^2+^ Tyrode’ solution and wild-type (yellow), STIM2β knockout cells (green) in 10 mM extracellular Ca^2+^ Tyrode’ solution (**F**) Histogram of basal calcium level of wild-type (black), STIM2β knockout cells (white). (n > 30 cells for each group) (**G**,**H**) (Edu staining of C2C12 wild-type cells (**G**) and STIM2β knockout cells (H) in 2 mM and 10 mM Ca^2+^ medium. (**I**) Histogram of Edu positive nuclei of wildtype cells and STIM2β knockout cells cultured in 2 mM or 10 mM Ca^2+^ medium. (n > 80 cells for each group) Error bars show means ± SEM. (All scale bars: 20 μm.) The results are representative of at least three independent experiments.

Since myogenesis is accompanied by decreased cell proliferation, the transition from proliferation to differentiation is an important and irreversible checkpoint of myogenesis^33,34^. Therefore, we investigated the effects of STIM2β knockout on the proliferation of C2C12 myoblasts using an MTT assay. After culture in growth medium for 3 days, the growth of the wild-type cells increased by 4-fold based on the change in the MTT-based OD value, while the STIM2β knockout cells increased by 8-fold (Fig. 4A). To confirm this result, equal numbers of cells were seeded on the growth medium and counted 72 h later. Consistently, STIM2β knockout cells showed 3-fold higher cell numbers than those in wild-type cells (Fig. 4B), confirming that STIM2β knockout promotes the proliferation of C2C12 myoblasts.

We next asked whether the increased proliferation of STIM2β knockout cells occurred because of abnormal intracellular Ca^2+^ homeostasis in STIM2β knockout cells. We examined STIM2β-dependent Ca^2+^ homeostasis by measuring the basal intracellular Ca^2+^ level according to the ratio of the intensity of the fluorescent dye Fura2-AM detected under plain media and growth media. The results showed that a significant increase in the cytosolic Ca^2+^ level in STIM2β knockout cells, which was then reduced with transient expression of the *STIM2*β gene in the knockout cells. These results implied that STIM2 regulates basal Ca^2+^ homeostasis of C2C12 myoblast cells (Figs 4C,D and S3A,B).

Furthermore, we performed EdU staining in standard Ca^2+^ concentration (2 mM) and high Ca^2+^ concentration (10 mM) growth media to clarify the relationship between high basal calcium level and increased proliferation rate of STIM2β knockout cells. Interestingly, both wild-type and STIM2β knockout cells had a significantly increased basal Ca2+ level and a fraction of EdU positive cells when grown in high Ca^2+^ concentration (10 mM) compared to that under a standard Ca^2+^ concentration (2 mM). Moreover, the STIM2β knockout cells showed a tendency to be more sensitive to the extra cellular Ca^2+^ concentration (Figs 4E–I and S3C,D). Hence, the increase in the intracellular Ca^2+^ concentration in the absence of STIM2β likely contributed to the increased proliferation of STIM2β knockout cells.

### STIM2β is involved in cyclin D1–CDK4-mediated cell-cycle arrest

**Figure 5:**
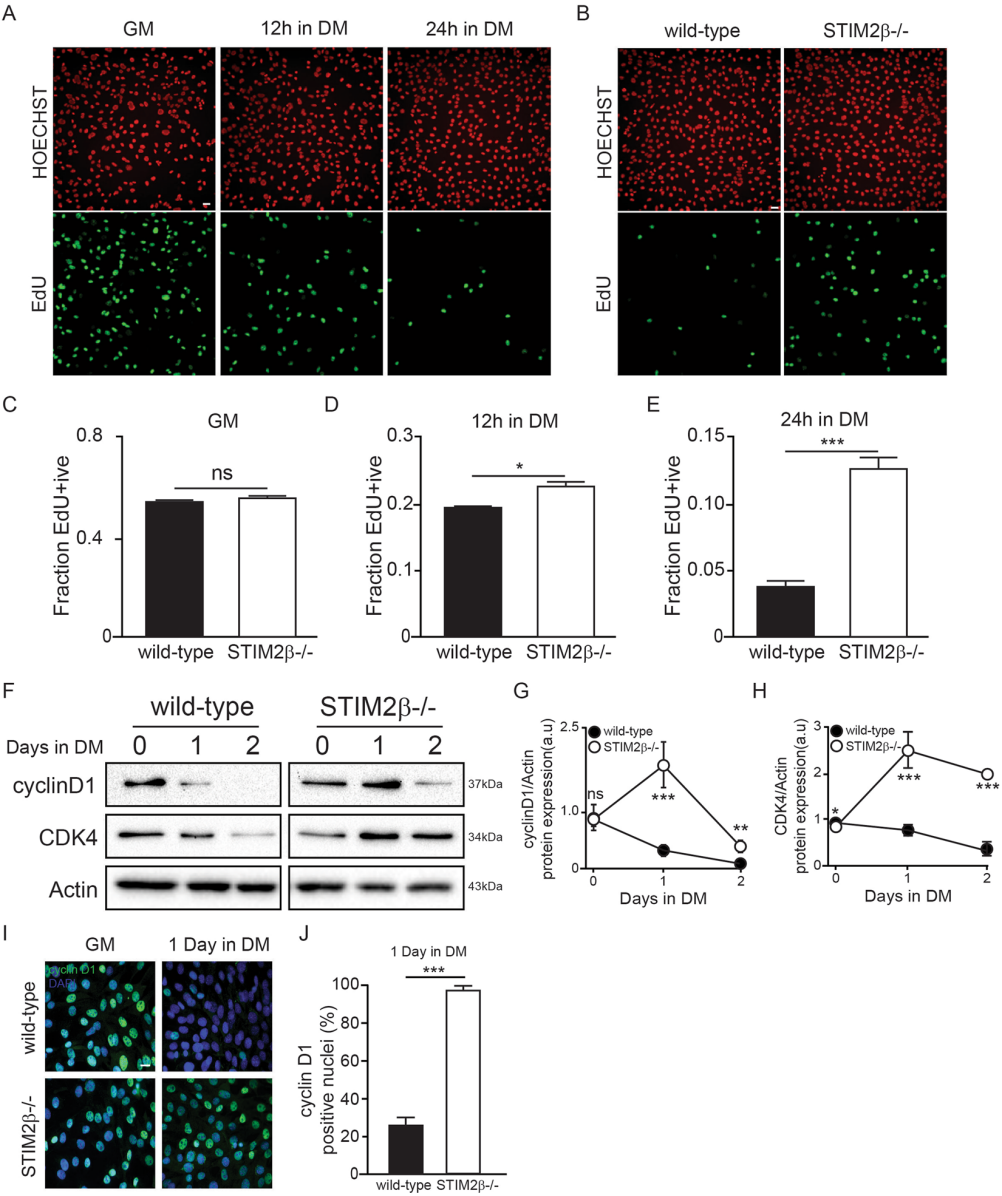
STIM2β knockout inhibited CyclinD1-CDK4 mediated cell cycle arrest. (**A**,**B**) Edu staining of C2C12 wild-type and STIM2β knockout cells. (**C**–**E**) Histogram of Edu positive nuclei of wild-type and STIM2β knockout cells in GM (**C**), 12 h in DM (**D**), and 24 h in DM (**E**). (n > 80 cells for each group) (**F**–**H**) Immunoblot analysis of wild-type cells and STIM2β knockout cells to measure the protein expression of CyclinD1 and CDK4 during myogenesis. Actin-normalized expression levels of CyclinD1 (**G**) and CDK4 (**H**) of wild-type cells (Black circle) and STIM2β knockout cells (White circle). (**I**,**J**) Immunofluorescence analysis of cyclin D1 expression in GM and DM 24 h (**I**) and relative quantification reporting the cyclin D1-positive nuclei percentage (**J**). (n > 130 cells for each group) Error bars show means ± SEM. (All scale bars: 20 μm.) The results are representative of at least three independent experiments.

To gain insight into the regulatory mechanism of STIM2β in the proliferation and differentiation process, we investigated the S-phase cells population based on EdU staining patterns. Wild-type cells rapidly withdrew from the cell cycle after myogenesis was induced (Fig. 5A–E), whereas this did not occur in STIM2β knockout cells, rapidly leading to a significantly large S-phase population at 12 and 24 h after inducing myogenesis (Fig. 5B–E). These results suggest that STIM2β regulates cell-cycle arrest during myogenesis.

Therefore, we next examined the expression of cell-cycle regulatory genes that control arrest (i.e., the G1–G0 transition). The mRNA expression levels of cyclin family genes showed similar patterns between wild-type and STIM2β knockout cells (Fig. S4). However, interestingly, when we checked the protein expression level of cyclin D1 and CDK4 that regulate cell-cycle arrest during myogenesis, wild-type cells showed decreased protein levels of cyclin D1 and CDK4 during myogenesis, while STIM2β knockout cells showed a 4–5 fold higher expression of cyclin D1 and 3–5 fold of CDK4 protein compared to wild-type cells (Fig. 5F–H), indicating that cell cycle arrest induced myogenesis might be delayed due to the altered the protein stability of cyclin D1. However, further study is needed how the protein stability of cyclin D1 is altered in STIM2β knockout cells. To further interrogate whether the stable protein expression of cyclin D1 in STIM2β knockout cells could induce the transcriptional activity of cyclin D1, we measured cyclin D1 positive nuclei portion. After inducing 1 day differentiation, only 25% of wild-type cells showed nuclear location of cyclin D1, compared with 97% of STIM2β knockout cells (Fig. 5I,J).

Taken together, these results implied that the STIM2β is required for the degradation of cyclinD1/CDK4 and cyclinD1/CDK4-mediated cell cycle arrest during skeletal muscle differentiation. Also, STIM2β dependent Ca^2+^ homeostasis regulates proliferation of myoblast cells, indicating that increased expression of STIM2β during myogenesis regulates transition of C2C12 cell-state from proliferation to differentiation.

## Discussion

STIM2β is considered to be a unique inhibitor of SOCE among all known STIM isoforms. A small increase of STIM2β can inhibit Orai function by forming heterodimer with STIM. Despite the fact that this feature was predicted to have an important effect on STIM2β during the development process, the exact underlying molecular mechanism is still unclear. In the present study, we found that the STIM2β signaling pathway plays a critical role in myogenesis. Our results demonstrate that STIM2β mRNA expression level was dramatically increased during early myogenesis. Knockout of STIM2β in C2C12 myoblast cells using the CRISPR/Cas9 technique show delayed myogenesis and increased proliferation process. Most importantly, we found that STIM2β regulates myogenesis by activating the MEF2C and NFAT4 signaling pathway, and promotes cell cycle arrest by decreasing cyclin D1 and its catalytic partner CDK4. Further, STIM2β reached the highest mRNA expression level at 6 days after inducing differentiation. Also, STIM2β knockout cells exhibited delayed myogenesis even at 7 days after inducing differentiation and showed impaired multinucleated muscle fiber formation, indicating that STIM2β might be required for not only early myogenesis but also later myogenesis (Fig. S5).

The effect of Ca2+ signaling on cell proliferation has been well documented^35^. Particularly, SOCE is the most extensively studied Ca2+ ignaling involved in cell proliferation. NFAT and CaM stimulation by SOCE is involved in the proliferation of various cell types and G1/S transition^36–41^. A previous study suggested that SOCE was relevant only in the first 12 h after the onset of C2C12 myogenesis and became less important in subsequent stages of the transition to mature muscle tissue differentiation^17^. These findings agree with those of other studies suggesting that myogenesis is accompanied by a transition between the proliferation and differentiation processes^33,34^. However, other studies have revealed that STIM and Orai are both strongly expressed in mature skeletal muscle cells and remain fully functional^42–44^. In this discrepancy, our results suggested that STIM2β is involved in SOCE through Orai1 channel during myogenesis (Fig. S6A). And confirmed by puncta formation of STIM2β with STIM1 and weakly recruited by Orai1 (Fig. S6 C). Also, STIM2β knockout cells showed increased SOCE amplitude and sustained SOCE peak compared with wild-type cells even after inducing differentiation (Fig. S6A,B). These results suggest that myoblast cells efficiently regulate SOCE via STIM2 alternative splicing during myogenesis.

In this study, we found that STIM2β is essential for intracellular Ca^2+^ signaling in myogenesis. This finding is inconsistent with a previous study suggesting that STIM2β did not have a significant effect on basal [Ca^2+^]_i_^25^. This discrepancy might suggest that around 40% expression level of STIM2β in naïve CD4 + T cells enough to regulate the basal intracellular Ca^2+^. Consistently, our results showed low expression of STIM2β in C2C12 myoblasts was still enough to regulate the basal intracellular Ca^2+^ level. Interestingly, the proliferation of C2C12 myoblasts was susceptible to changes in Ca^2+^ levels, and this phenomenon was more pronounced in STIM2β knockout cells. Moreover, the increased basal Ca^2+^ level of STIM2β knockout cells and requirement for STIM2ββ-dependent Ca^2+^ signaling for fine-tuning the proliferation of C2C12 myoblasts suggests that STIM2β regulates basal Ca^2+^ homeostasis in C2C12 myoblast cells. In this study, we suggest that STIM2β appeared to function as the key regulator of Ca^2+^ homeostasis in C2C12 myoblasts, and increased STIM2β expression induced a transition from proliferation to differentiation during myogenesis.

Given its high evolutionary conservation and widespread expression, the importance of STIM2β in various physiological functions is increasingly becoming recognized^26^. Moreover, the generation of STIM2β through alternative splicing is a particularly useful mechanism for modulating SOCE. Thus, the present insight of the role of STIM2β in myogenesis further highlights alternative splicing as an effective way to modulate the SOCE during development. Based on our findings, further investigation will be necessary to clarify the importance of STIM2β not only in skeletal muscle development but also in the developmental processes of other tissues and other physiological functions.

In summary, the present study demonstrates a previously unrecognized mechanism by which the STIM2β signaling pathway controls myogenesis. Under normal skeletal muscle development condition, the STIM2β positively regulates myogenesis by controlling cell-cycle arrest through regulating the degradation of cyclin D1-CDK4. Increased STIM2β will induce inhibition of SOCE during myogenesis to block its action on cell proliferation, which efficiently controls the transition of cell fate from proliferation to differentiation. At the same time, We further highlight that STIM2β activates myogenic factors through the MEF2C and NFAT4 pathway. Our findings contribute important insight toward gaining a better understanding of how myoblasts effectively regulate various signaling pathways during myogenesis processes through the alternative splicing of STIM2.

## Material and Methods

### Cell culture and transfection

C2C12 cells were cultured in Dulbecco’s Modified Eagle’s Medium (DMEM) supplemented with 10% fetal bovine serum (FBS) at 37 °C in 5% CO2. For the muscle cell differentiation, C2C12 myoblast cells were cultured to 70–80% confluency and then change cultured media to DMEM containing 2% horse serum. We changed the medium every day. For transient transfection, the cells were transfected at 70% confluency with 0.3–3 μg DNA using Jet Prime (PolyPlus) according to the manufacturer’s instructions.

### Intracellular Ca^2+^ imaging

Cells were loaded with 5 μM Fura-2/AM in DMEM at 37 °C for 30 min. Ratiometric Ca^2+^ imaging was performed at 340 and 380 nm in 2 mM Ca^2+^ Tyrode’s solution (129 mM NaCl, 5 mM KCl, 2 mM CaCl2, 1 mM MgCl2, 30 mM glucose, and 25 mM Hepes, pH 7.4) or cultued medium with a IDX81 microscope (Olympus) equipped with an Olympus x40 oil (NA 1.30) objective equipped with a fluorescent arc lamp (LAMDA LS), excitation filter wheel (SUTTER, LAMBDA 10–2), stage controller (ASI, MS-2000) and a CCD camera (HAMAMATSU, C10600) at room temperature. Images were processed with Metamorph and analyzed with Igor Pro.

### Reagents

Fura-2/AM was obtained from Invitrogen. Jet Prime was from Poly Plus. Thapsigargin (TG) was from Santa Cruz. Antibodies of myogenin(F5D, DSHB), MHC(MF-20, DSHB), CyclinD1(A-12, Santa Cruz), CDK4(B-10, Santa Cruz), β-actin(C4, Santa Cruz), MEF2C(MAB6786, R&D Systems) were purchased from the indicated vendor.

### mRNA expression level check

Total RNA was extracted from cells using RiboEx (GeneAll) following the manufacturer’s protocol. cDNA was made from 2~3 μg of RNA reverse transcribed using oligo (dT) primers and First Strand cDNA Synthesis Kit (TOYOBO). The PCR amplification was done using a C1000 Touch thermal Cycler (Bio-Rad). Amplification started with initial denaturation at 95 °C for 3 min and then 30–40 cycles of denaturation at 95 °C for 30 sec, annealing at 55 °C for 30 sec, and extension at 72 °C for 1 min. Gel electrophoresis was used to identify the PCR products in a 1.5% agarose gel using ethidium bromide staining. For real-time quantitative PCR analysis, synthesized cDNA and SYBR green Master Mix (Roche) were run on a LightCycler480 II (Roche). Relative expression levels of mRNA were calculated using the 2 ^−^ Ct method^45^.

### Western blotting

Cell lysates in lysis buffer (100 mM Tris-HCl, pH 8, 150 mM NaCl, 1% Triton X-100) were subjected to SDS-PAGE and electro-transferred onto PVDF membranes. The PVDF membrane was blocked with 7% Skim milk dissolved in Tris-buffered saline containing 0/1% Tween 20 for two h at room temperature with gentle shaking. After blocking, PVDF membrane was probed overnight at 4 °C, with specific primary antibodies in 3% BSA solution. The membrane was incubated for 30 min at RT with a horseradish peroxidase-conjugated anti-mouse or rabbit IgG in TBST. Detection was performed with the enhanced chemiluminescence reagent (ECL). Quantification of bands was performed by using the ImageJ software.

### Generation of knockout cell line with CRISPR/Cas9

Guide RNA sequences for mouse STIM2β (5′-CCTGCAGGTTAGTAGTTACTAGA-3′) are inserted in pRGEN vector and pRGEN-reporter (ToolGen). C2C12 cells were transfected with pRGEN-mSTIM2β, pRGEN-Cas9, and pRGEN-reporter using Lipofectamine 2000, according to the manufacture’s instructions. Two days after transfection, cells were selected with 1000 μg/ml of hygromycin for four days. After one week, colonies were an isolated and genomic deletion, and mRNA depletion was analyzed with sequencing and RT-PCR assay.

### Immunocytochemistry

C2C12 cells were seed on Collagen coated glass and induce differentiation. Every indicated timepoint, the cells were fixed with 4% paraformaldehyde for 10 min and treated with 0.1% Triton X-100 in PBS for 10 min at room temperature, and then block with 3% BSA in PBS for 2 hr. Following incubation with the indicated antibody over night at 4 °C, cells were incubated with a conjugated secondary antibody at 37 °C for 1 hr. Finally, the nuclei were stained with Hoechest (Molecular Probes) for 5 min.

### EdU staining

Cell-cycle analysis was performed by assaying the incorporation of 5-ethynyl-2′-deoxyuridine (EdU) according to the manufacturer’s instruction (Invitrogen). Briefly, cells were cultured in medium containing 1X EdU component for an additional time, as indicated. Following incubation, the cells were rinsed with PBS and fixed with 4% PFA for 15 mins at RT.

### MTT assay

C2C12 cells were seeded at a density of 7500 cells/well in 96-well plates. Next day, cells were incubated with medium containing 1 mg/ml of MTT. After 4 hr incubation at 37 °C, The medium was then aspirated and add DMSO and gently shake 15 min for formazan solubilization. The absorbance was measured at a wavelength of 562 nm by using a fluorescence plate reader (Spectra MAX Pro 5, Molecular Devices).

### Statistics

All statistical analysis was performed in Prism 6 (GraphPad Software). All error bars represent SEM. All pairwise differences were tested for significance using a two-tailed *t* test. At least three independent experiments were performed. P-values < 0.05 were considered statistically significant and indicated as following: *P < 0.05; **P < 0.01; ***P < 0.001.

## Supplementary information

Supplementary Information

## Data Availability

All data generated or analysed during this study are included in this published article (and its Supplementary Information files), are available from the corresponding author on reasonable request.
