# Supplementary material for: Orai1 inhibitor STIM2β regulates myogenesis by controlling SOCE dependent transcriptional factors
Source: Sci Rep. 2019 Jul 25;9:10794. doi: 10.1038/s41598-019-47259-5 (PMC6658661; doi:10.1038/s41598-019-47259-5)

**Orai1 inhibitor STIM2β regulates myogenesis by controlling SOCE dependent transcriptional factors**

Kyu Min Kim^1,3,*^, Anshul Rana^2,3^, Chan Young Park^1,*^

^1^Department of Biological Sciences, School of Life Sciences, UNIST, Ulsan, 44919, Republic of Korea.

^2^Department of Molecular and Cellular Physiology, Stanford University School of Medicine, Stanford. CA 94305.

^3^These authors contributed equally to this manuscript.

*Correspondence: K.K.M (email: kyumin4120@unist.ac.kr) and C.Y.P (email: cypark@unist.ac.kr)

**Supplementary Information (Materials and Methods)**

**Primers**

For mRNA expression level check, we use below indicated primers.

GAPDH

Forward : 5’-TGCACCACCAACTGCTTAG-3’

Reverse : 5’-GATGCAGGGATGATGTTC-3’

STIM1

Forward : 5’-TGGCCTGGGATCTCAGAGGG-3’

Reverse : 5’-TGTCCCCAACTGGAGATGGTGTG-3’

STIM2

Forward : 5’-AAGATCTGTGGCTTTCAGATAGC-3’

Reverse : 5’-AGTCACATTCTGAAGCTGTGTCTGG-3’

STIM2α

Forward : 5’-GCTAGCCATCGCTAAGGACGAGGCAG-3’

Reverse : 5’-AGCTATCTGAAAGCCACAGATCTTCTC-3’

STIM2β

Forward : 5’-TCGCTGCCTCCTATCTCCTGCAGG-3’

Reverse : 5’- AGCTATCTGAAAGCCACAGATCTTCTC-3’

Orai1

Forward : 5’-CCTGCATCCTGCCCAACATCGAGGC-3’

Reverse : 5’-TCCCCTCTGTGGTCCAGCTGGTCC-3’

Orai2

Forward : 5’-GGCCATGGTGGAGGTGCAGCTGGAG-3’

Reverse : 5’-ACCATGATGATGGTGGACACCAG-3’

Orai3

Forward : 5’-TGGGTCAAGTTTGTGCCCATTGG-3’

Reverse : 5’-CACAGCCTGCAGCTCCCCCTGC-3’

NFAT4

Forward : 5’-ACCAAAGCCTGGCCACACCC-3’

Reverse : 5’-CTCACTCACTTCCTCCAGGGTGAT-3’

CyclinD

Forward : 5’-TTGTGCCATCCATGCGGA-3’

Reverse : 5’-AAAGAAAGTGCGTTGTGCGG-3’

CyclinE

Forward : 5’-AAGCGAGGATAGCAGTCAGC-3’

Reverse : 5’-CGCTGCAGAAAGTGCTCATC-3’

CyclinA

Forward : 5’-GTCAACCCCGAAAAACTGGC-3’

Reverse : 5’-TGGCCCTCATGCTGTTAGTG-3’

CyclinB

Forward : 5’-AGTGCCTCTGAAAAGGGAAG-3’

Reverse : 5’-CTTCCTCCAGTTGTCGGAGA-3’

Myogenin

Forward : 5’-CTAAAGTGGAGATCCTGCGCAGC-3’

Reverse : 5’-GCAACAGACATATCCTCCACCGTG-3’

MHC

Forward : 5’-AGGGAGCTTGAAAACGAGGT-3’

Reverse : 5’-GCTTCCTCCAGCTCGTGCTG-3’

MEF2C

Forward : 5’-ATCTCTCCCTGCCTTCTACTC-3’

Reverse : 5’-CTCCCATCGTAGGAACTGCT-3’

For Orai1 R91W

Top : 5’-CTTAAAGCCTCCAGCTGGACCTCGGCTCTG-3’

Bottom : 5’- CTTAAAGCCTCCAGCTGGACCTCGGCTCTG-3’

STIM2β knockout check primer

Forward : 5’-GTAGTCTCTTATTTCATGATCAAT-3’

Reverse : 5’-TATCAGCCAAGATACAGACATGTT-3’

sh-STIM2β target sequence

5’-TCCTATCTCCTGCAGGCAGAA -3’


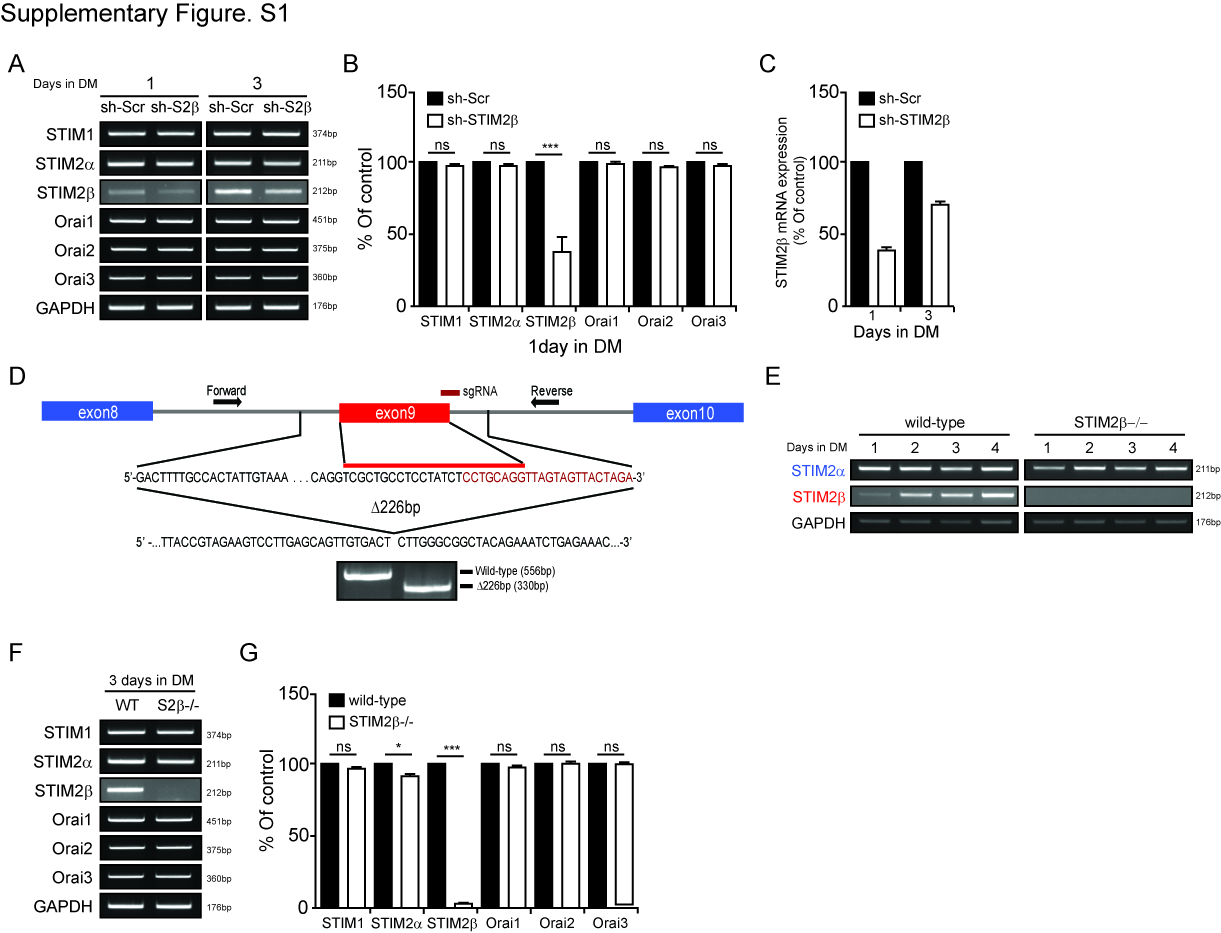


**Supplemental Figure S1. Specific knocdown and knockout of STIM2β.**

(A) Expression of other SOCE components during the differentiation of sh-Scramble and sh-STIM2β treated cells. (B) Relative expression of other SOCE components after transfection with sh-STIM2β. (C) Relative expression of STIM2β at 1day and 3day after inducing differentiation which transfection with sh-STIM2β. (D) Scheme of CRISPR-Cas9 for generation of STIM2β knockout cells. STIM2β cells were deleted 226nt in intron8-exon9-intron9 region. (E) Expression of STIM2 splicing variants during the differentiation of wild-type and STIM2β knockout cells. (F) Expression of other SOCE components of wild-type and STIM2β knockout cells. (G) Histogram of relative expression of other SOCE components of wild-type and STIM2β knockout cells. Error bars show means ± SEM. The results are representative of at least three independent experiments.


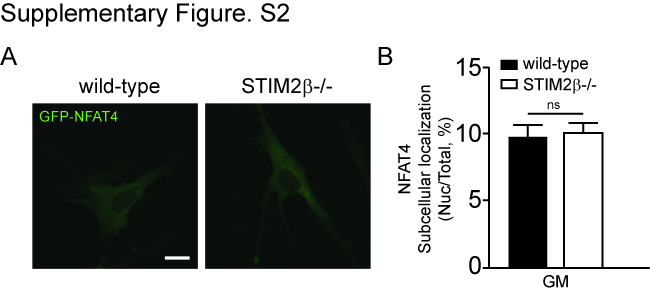


**Supplemental Figure S2. NFAT4 nuclear location at basal condition.**

(A) Subcellular distribution of GFP-NFAT4 in wild-type and STIM2β knockout cells. (B) Histograms of the mean abundance of nuclear-translocated NFAT4 under growth media cultured condition. (Scale bar : 20μm) Error bars show means ± SEM. The results are representative of at least three independent experiments.

**
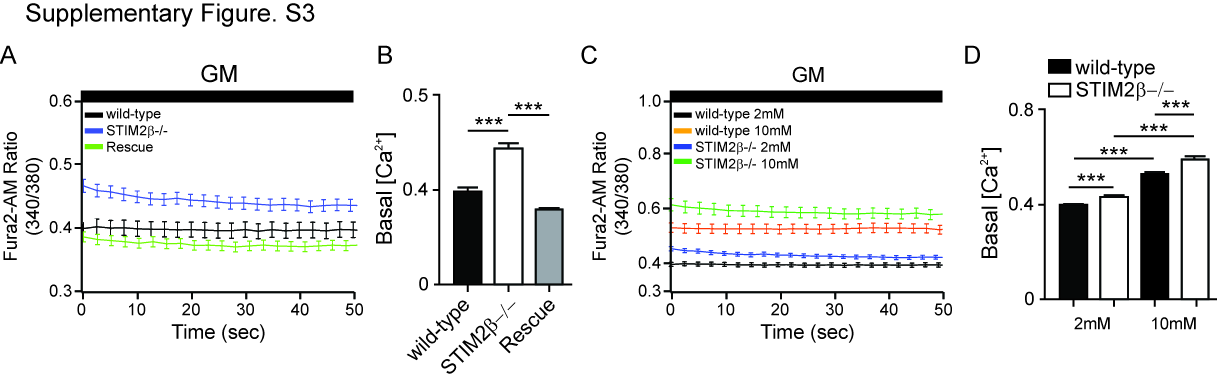
**

**Supplemental Figure S3. Basal [Ca^2+^]_i_ measurement of wild-type and STIM2β knockout cells.**

(A) Fura-2 Ca^2+^ measurements in growth media cultured condition. wild-type (black), STIM2β knockout cells (blue) and transiently STIM2β expressed in STIM2β knockout cells (green). (B) Histogram of basal calcium level of wild-type (black), STIM2β knockout cells (white) and transiently STIM2β expressed in STIM2β knockout cells (gray). (n > 30 cells for each group) (C) Fura-2 Ca^2+^ measurements. wild-type (black), STIM2β knockout cells (blue) in 2mM extracellular Ca^2+^ Tyrode’ solution and wild-type (yellow), STIM2β knockout cells (green) in 10mM extracellular Ca^2+^ Tyrode’ solution (D) Histogram of basal calcium level of wild-type (black), STIM2β knockout cells (white). (n > 30 cells for each group). Error bars show means ± SEM. The results are representative of at least three independent experiments.

**
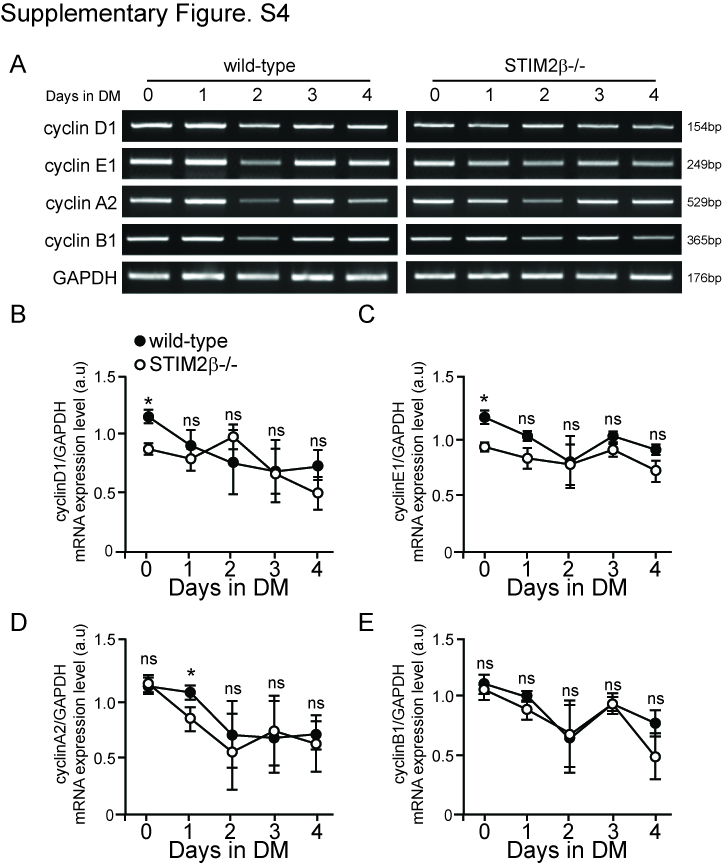
**

**Supplemental Figure S4. The mRNA expression level of cell-cycle regulator genes.** (**A**) mRNA expression level of cyclin family in wild-type (left) and STIM2β knockout cells (right). (B – E) Histogram of the cyclin D1 (B), cyclin E1 (C), cyclin A2 (D), cyclin B1 (E) mRNA expression level in wild-type (Black circle) and STIM2β knockout cells (White circle). Error bars show means ± SEM. The results are representative of at least three independent experiments.

**
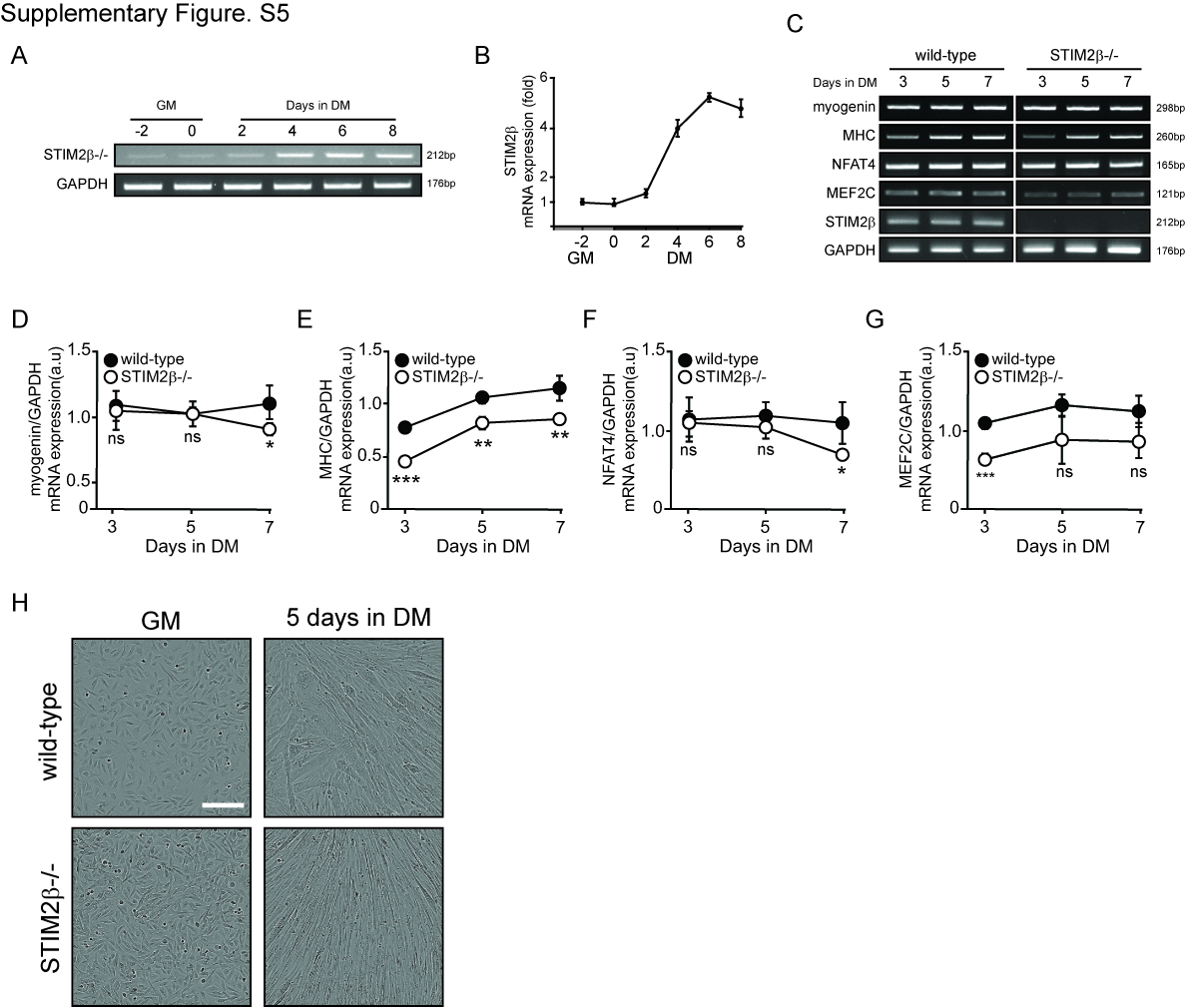
**

**Supplemental Figure S5. The role of STIM2β on later myogenesis.** (A) mRNA expression level of STIM2β in wild-type at later myogenesis. (B) Histogram of the STIM2β expression level. (C) Expression of myogenic factors during the differentiation of wild-type and STIM2β knockout cells. (D-G) GAPDH-normalized expression levels of myogenin (D), MHC (E), NFAT4 (F) and MEF2C (G). (H) Morphology of wild-type and STIM2β knockout cells in growth medium and 5days after inducing differentiation. (Scale bar : 200μm) Error bars show means ± SEM. The results are representative of at least three independent experiments.
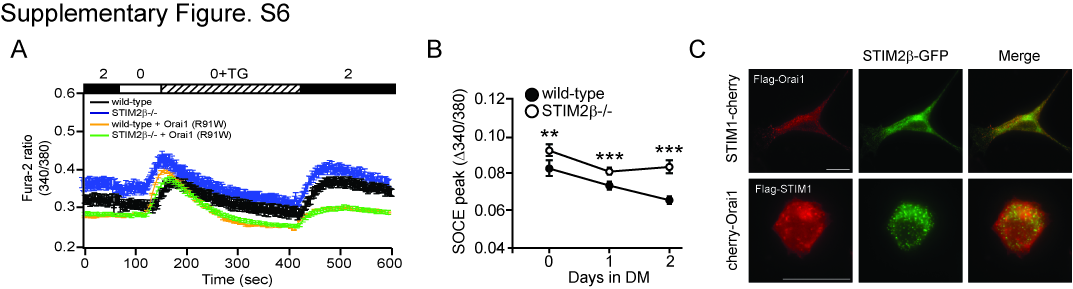


**Supplemental Figure S6. STIM2**β **is involved in SOCE during early myogenesis** (A) Fura-2 Ca2+ measurements in wild-type (black), STIM2β knockout cells (blue) and transiently Orai1 (R91W) expressed in wild-type (yellow) and STIM2β knockout cells (green). (B) Comparison of SOCE peaks during myogenesis in wild-type (black circle) and STIM2β knockout cells (white circle). (C) Localization of STIM2β-GFP with Cherry tagged STIM1 (upper panel) or Orai1 (bottom panel) with TG treated condition. (All scale bars : 20μm) Error bars show means ± SEM. The results are representative of at least three independent experiments.

**Full length blots/gels**


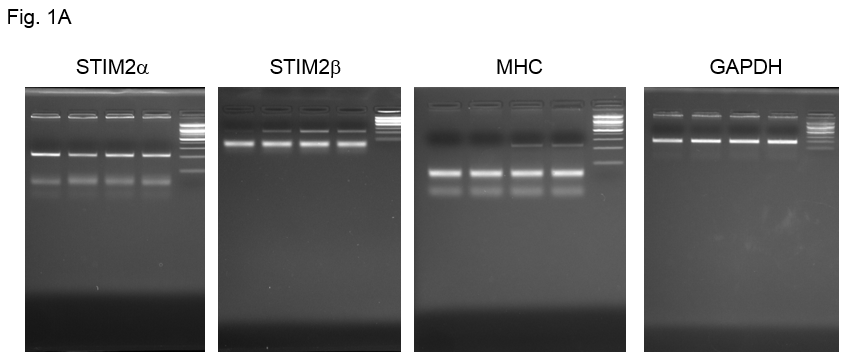


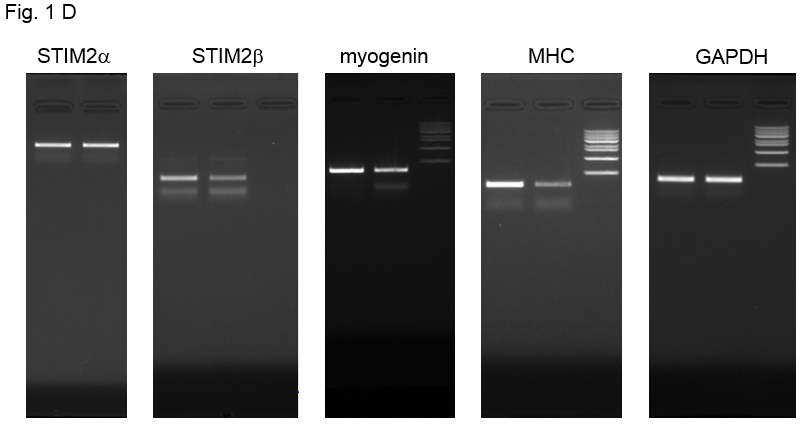


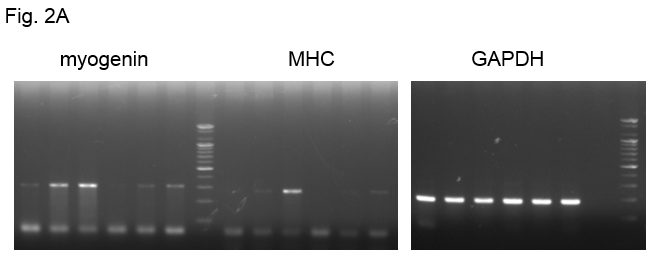


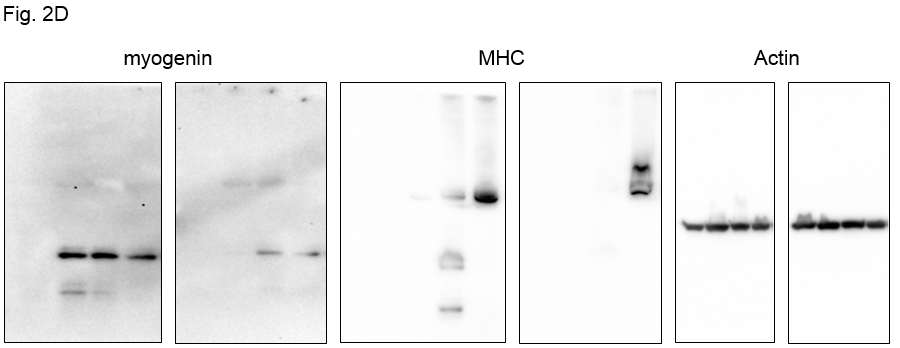


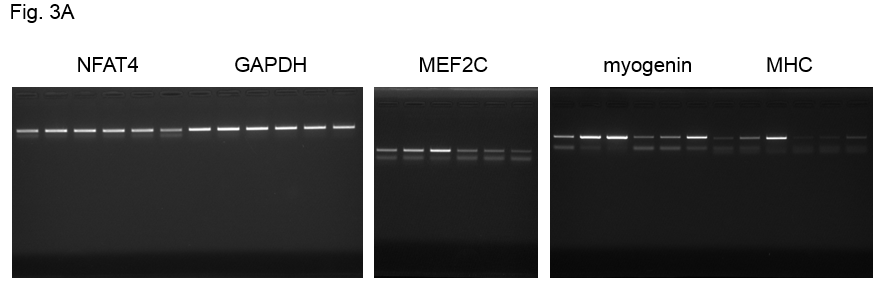


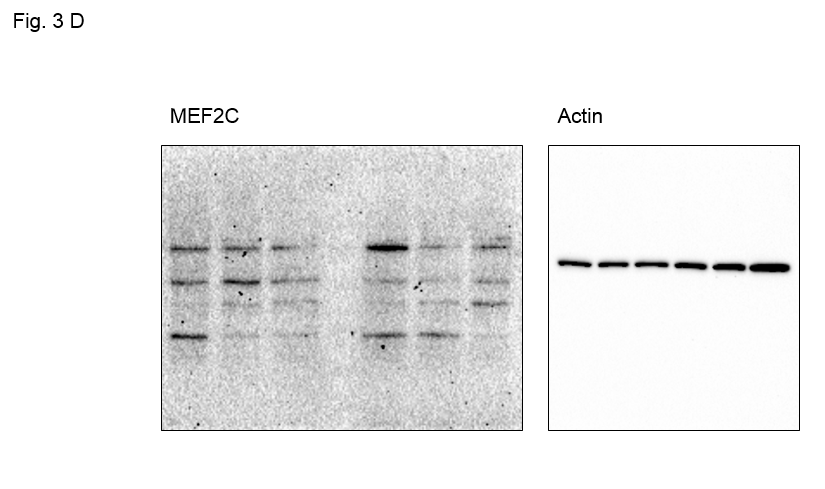


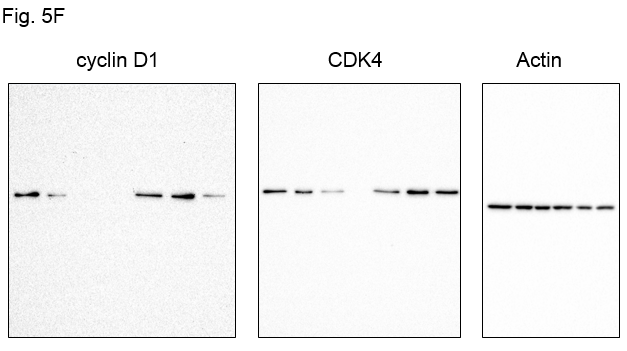


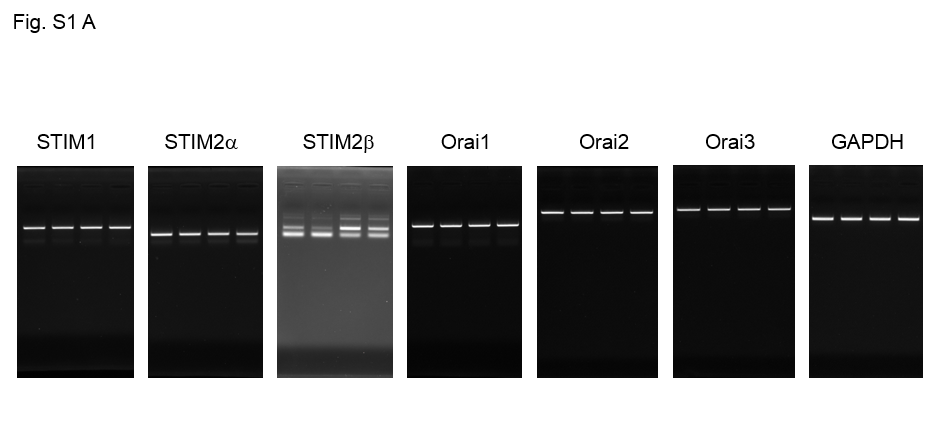


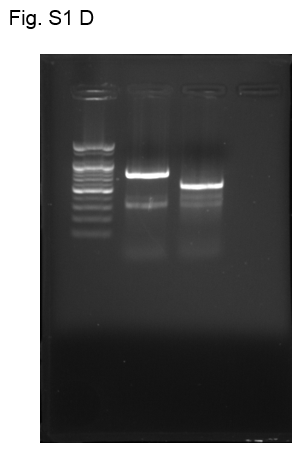


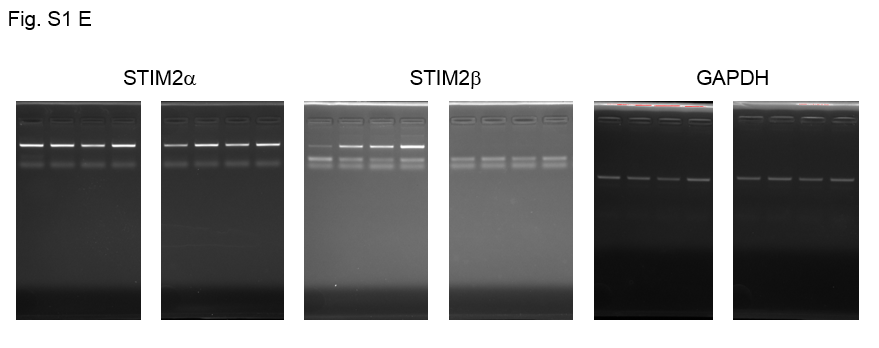


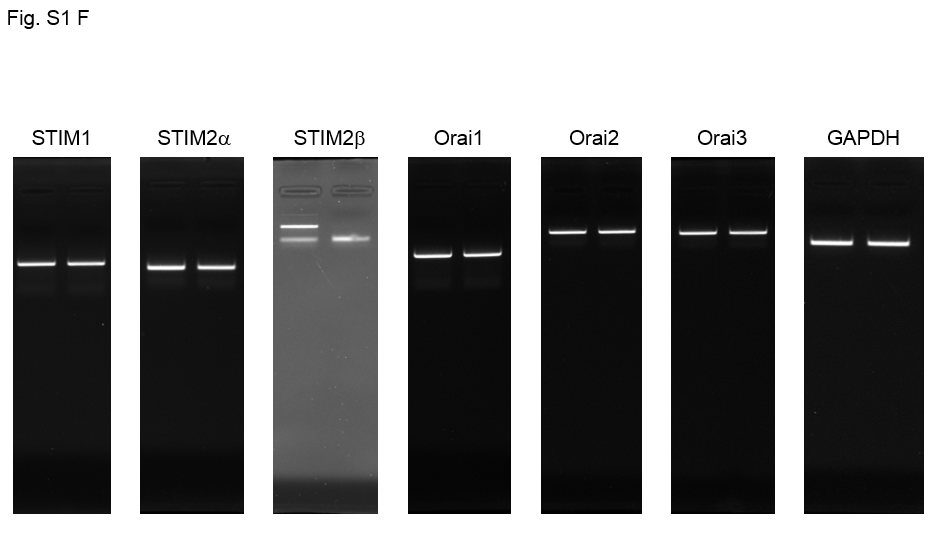


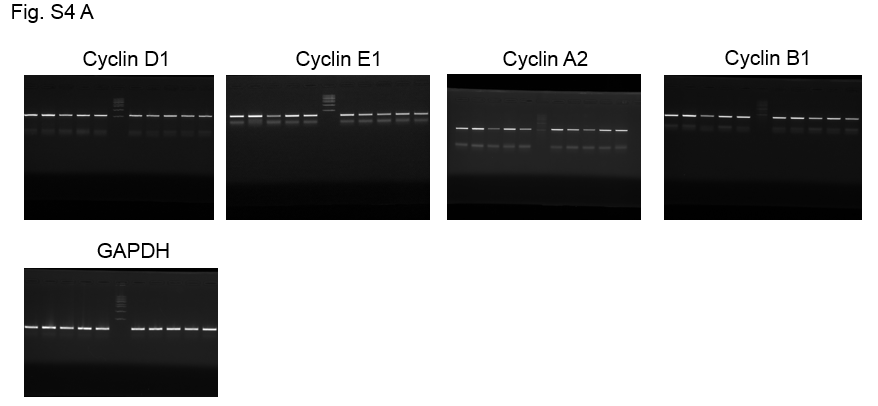


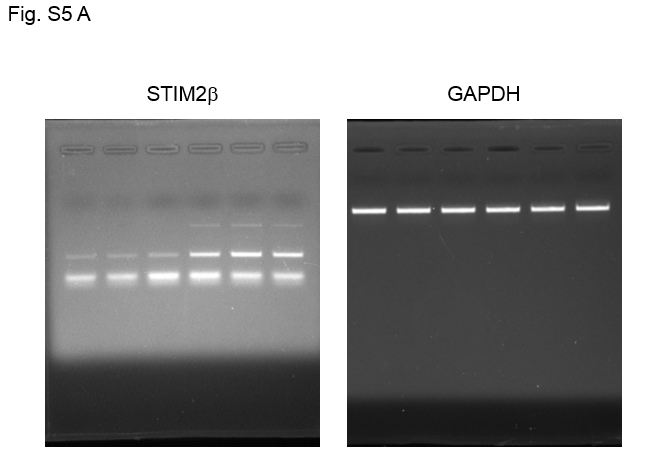


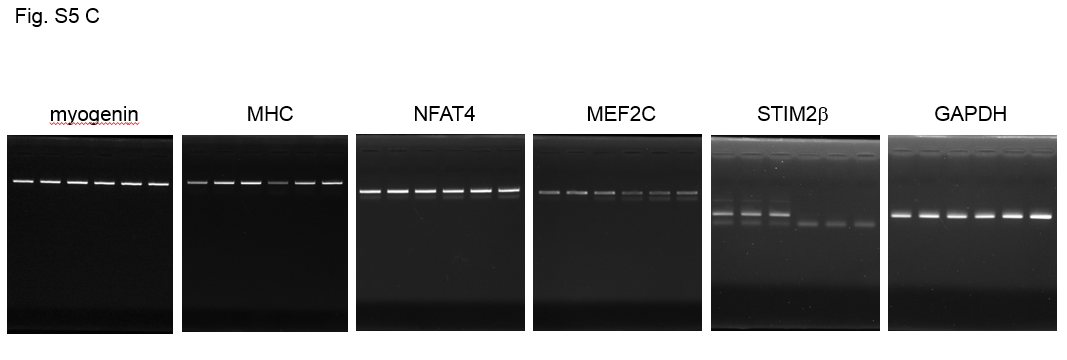

Supplement: Supplementary file 1 — Supplementary Information [file 41598_2019_47259_MOESM1_ESM.docx]
